# J. Branson's, Esq. Surgeon, Letter to Dr. Pearson

**Published:** 1801-02

**Authors:** J. Branson

**Affiliations:** Doncaster


### J. Branson's, Esq. Surgeon, Letter to Dr. Pearson.

Dear Sir,

In my laft letter to you, I mentioned my ideas refpecting the infufceptibility to the Vaccine Contagion in a perfon who had previoufly had the Small-pox. I am ftill of the fame opinion, *as I have never yet been able to produce the true vaccine puftule in a perfon who had before undergone the Small-pox.* But I have lately had a Cafe, from which it would appear, that if both difeafes are introduced into the fyftem about the fame time, that they will both be received, and proceed through their regular ftages, without being at all affected by each other. — A child was inoculated for the Cow-pock after it had been for fome days expofed to the contagion of the natural Small-pox, on Friday, the 26th of September. The arm went on in the ufual way, until the Wednefday evening following, when the child was feized with fever; and in the evening of the next day the Small-pox appeared. The eruption was confluent, and particularly fo about the inoculated part. On Monday the 6th of October, which was the eleventh day from the infertion of the Vaccine matter, and the fifth from the commencement of the fever of the Small-pox, I inoculated a child from the Cow-pock puftule, furrounded by a clufter of Small-pox; in fhort, they appeared fo blended, that I had my doubts whether the matter I obtained was that of the Cow-pock, or mixed with the Small-pox: Indeed, I was rather difpofed to think the latter was the cafe, as it did not appear fo limpid as it ufually does in the Cow-pock. However, the refult was, that it produced the genuine Cow-pock, without any appearances different from what I have always feen; and with matter from this fource I have continued to inoculate ever fince, without any variation either in fymptoms or appearance. If this Cafe furnifhes you with any thing new on the fubject, I fhall be very glad: I have no time now to make any comments upon it, as the Poft-office is juft going to fhut. I am,

Dear Sir,

Your's, fincerely,

J. BRANSON.

*Doncaster, Nov.* 23, 1800.

P. S. Since I wrote laft to you, I have inoculated upwards of five hundred for the Vaccine Difeafe, without any unpleafant fymptom.

